# Novel nano-fertilizers derived from drinking water industry waste for sustained release of macronutrients: performance, kinetics and sorption mechanisms

**DOI:** 10.1038/s41598-024-56274-0

**Published:** 2024-03-08

**Authors:** Samira S. Elsabagh, Elsayed A. Elkhatib, Mohamed Rashad

**Affiliations:** 1 Arid Lands Cultivation Research Institute, City of Scientific Research and Technological Applications, New Borg El-Arab, Alexandria 21934 Egypt; 2 Department of Soil and Water Sciences, Faculty of Agriculture (El-Shatby), Alexandria University, Alexandria, 21545 Egypt

**Keywords:** Water treatment residuals, Water retention, Adsorption mechanisms, Efficient release, Sustainable agriculture, Solid Earth sciences, Environmental monitoring

## Abstract

Nanotechnology has emerged as a promising approach for the controlled release of nutrients, particularly phosphorus and potassium. These essential plant nutrients are often applied in excess, leading to environmental pollution and loss of efficiency in crop production. Innovative economic and highly efficient fertilizers are urgently needed to achieve the targeted crop production worldwide in the presence of limited land and water resources. Therefore, in this study, novel, eco-friendly, cost-effective and enhanced efficiency nano-enabled fertilizers, NEF (nWTF1and nWTF2) were synthesized by impregnation of nanostructured water treatment residuals (nWTR) with (KH_2_PO_4_ + MgO) at 1:1 and 3:1 (w/w) ratios respectively using a planetary ball mill. The nWTR, nWTF1 and nWTF2 were extensively characterized. The water retention behavior and the sustained release of nutrients from the fabricated nano-enabled fertilizers (nWTF1 and nWTF2) in distilled water and sandy soil were investigated and monitored over time. The water retention capacity of the soil treated with nWTF2 after 26 days was 9.3 times higher than that of soil treated with conventional fertilizer. In addition, the nWTF2 exhibited lower release rates of P, K and Mg nutrients for longer release periods in comparison with the conventional fertilizers. This is a significant advantage over traditional fertilizers, which release nutrients quickly and can lead to leaching and nutrient loss. The main interaction mechanisms of PO_4_–K–Mg ions with nWTR surface were suggested. The results of the kinetics study revealed that power function was the best suitable model to describe the kinetics of P, K and Mg release data from NEF in water and soil. The produced NEF were applied to Zea maize plants and compared to commercial chemical fertilizer control plants. The obtained results revealed that the nano-enabled fertilizers (nWTF1 and nWTF2) significantly promoted growth, and P content compared with the commercial chemical fertilizer treated plants. The present work demonstrated the power of nano enabled fertilizers as efficient and sustained release nano-fertilizers for sustainable agriculture and pollution free environment.

## Introduction

World population is predicted to average 9.8 billion by the year 2050^1^. Thus, a significant demand of world agriculture production is anticipated. FAO^2^ predicted 70% increase in global production of grain by 2050 to cover such demand. To achieve the targeted crop production worldwide in the presence of limited land and water resources, a significant increase in agricultural fertilizer application is required. Phosphorus and potassium based fertilizers are essential plant nutrients that are commonly used to improve crop yields. Because traditional fertilizers are inefficient in delivering nutrients to plants, they are often applied in excess which eventually cause nutrients loss, eutrophication and serious environmental problems related to soil and water contamination^3,4^. To control these economic and environmental obstacles, innovative development of low-cost highly efficient fertilizers is needed to improve nutrients retention for optimal crop production and to minimize environmental disruptions of globally sustainable agriculture. The choice of utilization of cheap and nontoxic industrial waste materials for economic gain could provide environmental safety and sustainability to mankind and assist in maintaining soil quality^5,6^.

The use of innovative nanotechnology in agriculture (i.e. Nano-enabled fertilizers (NEF) development) is considered one of the most promising approaches to significantly slow and sustained release of fertilizer, increase crop production and prevent the loss of nutrients into the environment^7,8^. To address these challenges, nano-enabled fertilizers should be supplied to the plants over an extended period of time to noticeably reduce the fertilizer application rate. With the aid of ball milling technology, novel economic controlled release nanostructured fertilizers could be developed through incorporating a natural byproduct in nanoscale as a carrier to generate nano-enabled sustained release fertilizers having one or more nanoscale components^9–11^.The advantages of using eco-friendly nano-carriers are tuning the fertilizers to release nutrients in a controlled manner through the sustainable reuse of industrial byproducts sources^12–16^.

Water treatment residuals (WTRs) are waste products of drinking water industry, composed mainly from sediment, silica and iron salts—Fe_2_ (SO_4_)_3_- or aluminum salts (Al_2_(SO_4_)_3_ which are regularly used in coagulation, flocculation, and sedimentation processes for water purification^16^. Huge amounts of WTRs are produced yearly worldwide. The low-cost and eco-friendly WTRs have become popular due to its efficacy in removing contaminants^17–20^. An earlier research has demonstrated that WTRs in nanoscale considerably increase its active adsorption surface area and consequently increase its capacity for heavy metals adsorption. Elkhatib et al.^16^ successfully synthesized nanostructured WTRs sorbent with the aid of planetary ball mill and reported that very small percentage (< 3.5%) of P was released from P-saturated nWTRs demonstrating P stability in P-nWTR system. The excellent adsorption capability of nWTR for inorganic pollutants indicates its potential applicability as a carrier for plant nutrients. Data are not available, however, in the literature on using the low-cost efficient nWTRs as a carrier in the field of controlled release fertilizers.

To date, comparatively few nano- enabled fertilizers (NEFs) have been developed with little is known concerning their potential of agricultural application and safety^5–14^. Therefore, research is urgently needed to clarify ways to enhance resource efficiency through developing economic and natural NEFs. The main goal of this study was to produce eco-friendly, cost-effective and enhanced efficiency nano-enabled fertilizer based eco-friendly material (nWTRs) and evaluate its nutrients release pattern and kinetics. This research provides—for the first time-information for the potential use of nanostructured WTRs as a carrier to sustain plant nutrients release and to present an eco-friendly solution through the sustainable reuse of natural sources.

## Materials and method

### Preparation and characterization of nWTRs

**Figure 1:**
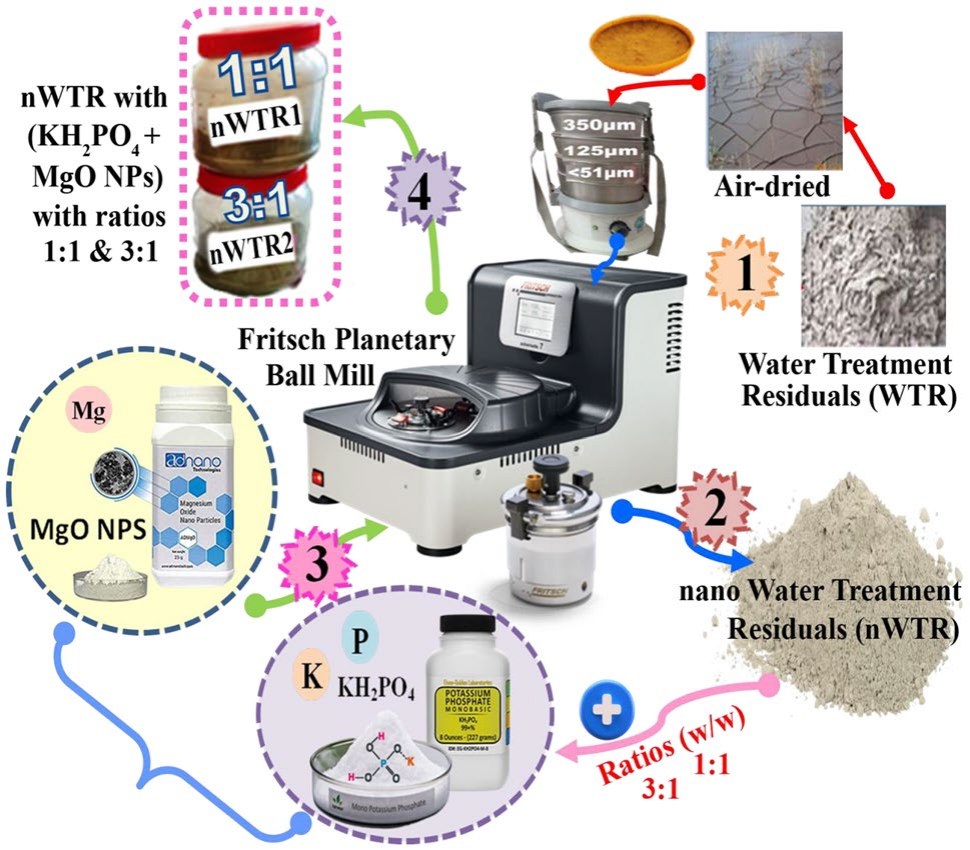
Schematic diagram of synthesis steps of nano- enabled fertilizers (NEF) based water treatment residuals nanoparticles (nWTR).

The collected WTRs were transported from drinking water treatment plant (Alexandria, Egypt) to the laboratory. The bulk WTRs was mechanically ground using a stainless steel hammer mill, passed into a 0.51 µm sieve to obtain the WTRs powder (Fig. 1).The WTR powder (15 g) was ground to the desired nanoscale following the method of Elkhatib et al.^12^ by using Fritsch Planetary Mono Mill Pulverisette 6 classic line equipped with 80-ml stainless steel grinding bowl and 150 g of 1 mm steel grinding balls (10). The milling operation was conducted by alternating 10 min of milling with 5 min of rest to avoid excessive heat. The nanostructured WTR (nWTR) carrier was stored in ziploc polyethylene bags until further use. Sizes, shape, surface morphology of nWTRs were explored by TEM (H-7650, Hitachi, Japan). The crystallography phase of nWTRs was characterized through X-ray diffractogram, XRD (Bruker D2 Phaser diffractometer) and the diffractogram was recorded in the 2θ range = 0–100°. The surface chemical structure of the produced samples was evaluated by X-ray photoelectron spectroscopy (XPS).

### Synthesis and characterization of nano-enabled fertilizer based nWTR

The conventional fertilizers (KH_2_PO_4_ and MgO, powder) used as P–K–Mg nutrients sources were of analytical grade and were obtained from Sigma-Aldrich Inc (Massachusetts, US). The nanostructured WTR (nWTR) carrier and the (KH_2_PO_4_ + MgO, powder) were mixed at 1^nWTR^: 1 ^KH^_2_^PO^_4 _^+MgO^ and 3 ^nWTR^: 1 ^KH^_2_^PO^_4 _^+MgO^ (w/w) ratios and placed in a planetary ball mill (Pulverisette-7, Fritsch, Germany) at 200 rpm for 30 min. The produced two impregnated nano-enabled fertilizers are referred to nWTF1 and nWTF2 respectively (Fig. 1). Scanning Electron Microscopy with Energy Dispersive X-Ray analyzer **(**SEM–EDX, INCAx-Sightmodel 6587, Oxford instruments, UK) was employed to identify the elemental composition and surface characteristics of the nano-carrier (nWTR) and the produced nano- enabled fertilizers (nWTF1 and nWTF2). The functional groups analysis of nWTR, nWTF1 and nWTF2 was performed by Fourier transform infrared spectroscopy (FTIR, Alpha, Bruker, Germany).

### Soil collection and analysis

A sandy soil (Typic torripsamment) was sampled from EL-Alamien, Alexandria, Egypt at sampling depth of 0–15 cm. The soil samples collected were air-dried, crushed, and sieved through 2 mm sieve. Soil physical and chemical characteristics were determined using standard methodology^21^. The measured soil properties are presented in supplemental materials (Table S2).

### Water-retention behavior of nWTF1 and nWTF2 in soil

The water retention behaviors of the nano-enabled fertilizers (nWTF1 and nWTF2) in sandy soil were determined following the method of Wei et al.^22^. The method in details is presented in supplemental materials.

### Release behavior of nWTF1 and nWTF2

The sustained release pattern of P, K & Mg nutrients from the fabricated nano-enabled fertilizers (nWTF1 and nWTF2) in distilled water and sandy soils were investigated^22,23^. The experiments are detailed in supplemental materials.

### Mathematical modeling of P, K and Mg release profiles

The P, K and Mg released data obtained from the NEF release experiments were fitted to four different kinetic models (first order, Elovich, Parabolic diffusion and power function) (Tables 1, 2 and 3) to investigate the release kinetics of P, K and Mg from water and soil amended with NEF and to pin point the best predictive model capable of describing the results^24–27^.

**Table 1:** Kinetics Models for P release from NEF in water and soil.

Models release kinetics	Description	Parameter	Water			Soil		
			nWTF1	nWTF2	C	nWTF1	nWTF2	C
First order ${\text{Ln}}\left( {{\text{q}}_{0} - {\text{q}}_{{\text{t}}} } \right) = {\text{a}} - ({\text{K}}_{{\text{d}}} *{\text{T}})$	q_0_ = amount of P released at equilibrium mg g^−1^, q or q_t_ = amount of P released at time t (h) mg g^−1^, K_d_ = Solubility rate (h^−1^), a = constant (mg g^−1^)	K_d_	0.012	0.011	0.013	0.005	0.003	0.007
		a	5.451	4.393	6.442	2.215	1.026	3.738
		R^2^	0.923	0.913	0.942	0.983	0.975	0.967
		SE	0.218	0.199	0.206	0.07	0.038	0.151
Elovich ${\text{q}}_{{\text{t}}} = \left( {{1}/\upbeta } \right){\text{ln}}\left( {\alpha \upbeta } \right) + \left( {{1}/\upbeta } \right){\text{lnt}}$	α = initial desorption rate of P (mg/g h) β = constant related to P release (mg g^−1^)	α	14.14	4E+00	6E+01	0.102	2E−02	4E−01
		β	0.029	0.089	0.01	0.312	0.931	0.080
		R^2^	0.737	0.669	0.789	0.949	0.810	0.973
		SE	38.66	14.95	94.78	0.562	0.395	1.581
Parabolic diffusion ${\text{q}} = {\text{a}} + {\text{K}}_{{\text{d}}} {\text{t}}^{{{1}/{2}}}$	a = constant (mg g^−1^) K_d_ = apparent diffusion rate coefficient (mg/g h^1/2^)	K_d_	15.21	5.131	42.21	0.529	0.184	2.051
		a	29.43	13.50	36.07	1.865	1.336	7.405
		R^2^	0.945	0.905	0.969	0.991	0.908	0.998
		SE	17.66	8.015	36.03	0.233	0.273	1.489
Power function ${\text{logq}} = {\text{log K}}_{{\text{d}}} {\text{C}}_{o} + {1}/{\text{m logt}}$	K_d_ = apparent desorption rate coefficient(h^−1^) 1/m = constant Co = initial P concentration	K_d_	4.811	0.577	38.17	0.131	0.003	0.410
		1/m	0.682	0.868	0.492	0.705	1.117	0.743
		R^2^	0.978	0.978	0.964	0.995	0.983	0.993
		SE	0.089	0.115	0.078	0.017	0.049	0.021

C = Classical fertilizers (control).

**Table 2:** Kinetics Models for K release from NEF in water and soil.

Models release kinetics	Description	Parameter	Water			Soil		
			nWTR1	nWTR2	C	nWTR1	nWTR2	C
First order ${\text{Ln}}\left( {{\text{q}}_{0} - {\text{q}}_{{\text{t}}} } \right) = {\text{a}} - ({\text{K}}_{{\text{d}}} *{\text{T}})$	q_0_ = amount of K released at equilibrium mg g^−1^, q or q_t_ = amount of K released at time t (h) mg g^−1^, K_d_ = Solubility rate (h^−1^), a = constant (mg g^−1^)	K_d_	0.017	0.014	0.019	0.006	0.006	0.005
		a	6.631	6.230	7.352	3.741	2.791	5.583
		R^2^	0.969	0.990	0.908	0.907	0.950	0.942
		SE	0.198	0.089	0.399	0.196	0.139	0.151
Elovich ${\text{q}}_{{\text{t}}} = \left( {{1}/\upbeta } \right){\text{ln}}\left( {\alpha \upbeta } \right) + \left( {{1}/\upbeta } \right){\text{lnt}}$	α = initial desorption rate of K (mg/g h) β = constant related to K release (mg g^−1^)	α	96.28	6E+01	2E+02	0.240	1E−01	2E+00
		β	0.008	0.011	0.004	0.081	0.203	0.012
		R^2^	0.863	0.852	0.865	0.871	0.883	0.893
		SE	122.5	102.4	175.4	3.66	1.37	22.01
Parabolic diffusion ${\text{q}} = {\text{a}} + {\text{K}}_{{\text{d}}} {\text{t}}^{{{1}/{2}}}$	a = constant (mg g^−1^) K_d_ = apparent diffusion rate coefficient (mg/g h^1/2^)	K_d_	53.49	37.39	98.65	2.081	0.814	13.70
		a	6.514	11.36	6.99	13.83	4.313	79.07
		R^2^	0.983	0.984	0.990	0.918	0.918	0.962
		SE	33.98	22.77	46.84	2.921	1.145	12.81
Power function ${\text{logq}} = {\text{log K}}_{{\text{d}}} {\text{C}}_{o} + {1}/{\text{m logt}}$	K_d_ = apparent desorption rate coefficient(h^−1^) 1/m = constant Co = initial K concentration	K_d_	55.37	37.22	124.2	0.056	0.028	0.901
		1/m	0.487	0.490	0.449	1.168	0.903	0.902
		R^2^	0.976	0.976	0.985	0.979	0.967	0.993
		SE	0.064	0.064	0.046	0.006	0.055	0.024

**Table 3:** Kinetics models for Mg release from NEF in water and soil.

Models release kinetics	Description	Parameter	Water			Soil		
			nWTR1	nWTR2	C	nWTR1	nWTR2	C
First order ${\text{Ln}}\left( {{\text{q}}_{0} - {\text{q}}_{{\text{t}}} } \right) = {\text{a}} - \left( {{\text{ K}}_{{\text{d}}} *{\text{T }}} \right)$	q_0_ = amount of Mg released at, equilibrium mg g^−1^, q or q_t_ = amount of Mg released at time t (h) mg g^−1^, K_d_ = Solubility rate (h^−1^), a = constant (mg g^−1^)	K_d_	0.012	0.012	0.012	0.005	0.006	0.006
		a	5.503	5.172	5.967	3.193	2.605	3.178
		R^2^	0.991	0.956	0.975	0.938	0.876	0.911
		SE	0.077	0.167	0.129	0.159	0.257	0.207
Elovich ${\text{q}}_{{\text{t}}} = \left( {{1}/\upbeta } \right){\text{ln}}\left( {\alpha \upbeta } \right) + \left( {{1}/\upbeta } \right){\text{lnt}}$	α = initial desorption rate of Mg (mg/g h) β = constant related to Mg release (mg g^−1^)	α	31.07	3E+01	5E+01	0.144	6E−02	2E−01
		β	0.023	0.034	0.015	0.135	0.263	0.142
		R^2^	0.862	0.855	0.859	0.885	0.854	0.883
		SE	32.54	23.04	50.58	2.029	1.195	1.945
Parabolic diffusion ${\text{q}} = {\text{a }} + {\text{K}}_{{\text{d}}} {\text{t}}^{{{1}/{2}}}$	a = constant (mg g^−1^) K_d_ = apparent diffusion rate coefficient (mg/g h^1/2^)	K_d_	4.668	3.151	27.95	1.248	0.645	1.183
		a	18.09	12.48	6.495	8.344	5.178	6.577
		R^2^	0.989	0.984	0.993	0.962	0.943	0.961
		SE	8.871	7.698	11.47	1.175	0.748	1.127
Power function ${\text{logq}} = {\text{logK}}_{{\text{d}}} {\text{C}}_{o} + { 1}/{\text{m logt}}$	K_d_ = apparent desorption rate coefficient (h^−1^) 1/m = constant Co = initial Mg concentration	K_d_	18.33	17.03	29.70	0.028	0.001	0.110
		1/m	0.487	0.436	0.478	1.079	1.523	0.832
		R^2^	0.982	0.975	0.992	0.938	0.983	0.985
		SE	0.055	0.057	0.035	0.028	0.066	0.034

C = Classical fertilizers (control).

### Pot experiment

Pot experiment was conducted to estimate the effect of NEF on *Zea maize* growth and phosphorus content in plant. Three treatments with three replicates were performed in the pot experiment, comprising the C (control, the soil treated with P, K and Mg conventional fertilizers), nWTF1 and nWTF2. All treatments were received the same amount of phosphorus (150 mg kg^−1^). A sandy soil was air-dried, sieved (2 mm) and packed into pots (95 mm diameter, 50 mm deep), with a total of 500 g soil. Firstly 300 g of the sandy soil was placed in the pots. All treatments received nitrogen fertilizer (urea) at a rate of 60 mgN g^−1^ per pot. Then the fertilizer samples mentioned above were evenly spread on the top of the soil. The P, K and Mg contents of nWTR (nano-carrier) and sustained release fertilizers (nWTF1 and nWTF2) are presented in Table S4 (Supplementary materials)**.** After that, 200 g of soil was placed to cover the fertilizer samples. Finally three seedlings were placed in each pot. The plants were grown for 25 d before harvesting.

All plants were grown under greenhouse conditions and were watered with 50 mL of water (each pot) day after day. At the early seedling stage, the stem diameter and height of the plant were measured. At harvest (25 days), plant shoots and roots were weighed (fresh mass) and dried at 60 °C before being weighed again (dry mass). The tissues were then ground, digested in a 1: 3 mixture of nitric acid and hydrochloric acid, and analyzed. The P content in maize seedling was determined using the method reported by Reuter and Robinson^28^.

### Statistical analysis

All data were analyzed by using SPSS (23.0) statistics and Microsoft Excel. Different letters assigned to means indicate statistically significant differences (*P* ≤ 0.05).

## Results and discussion

### Characterization

#### XRD and TEM studies

**Figure 2:**
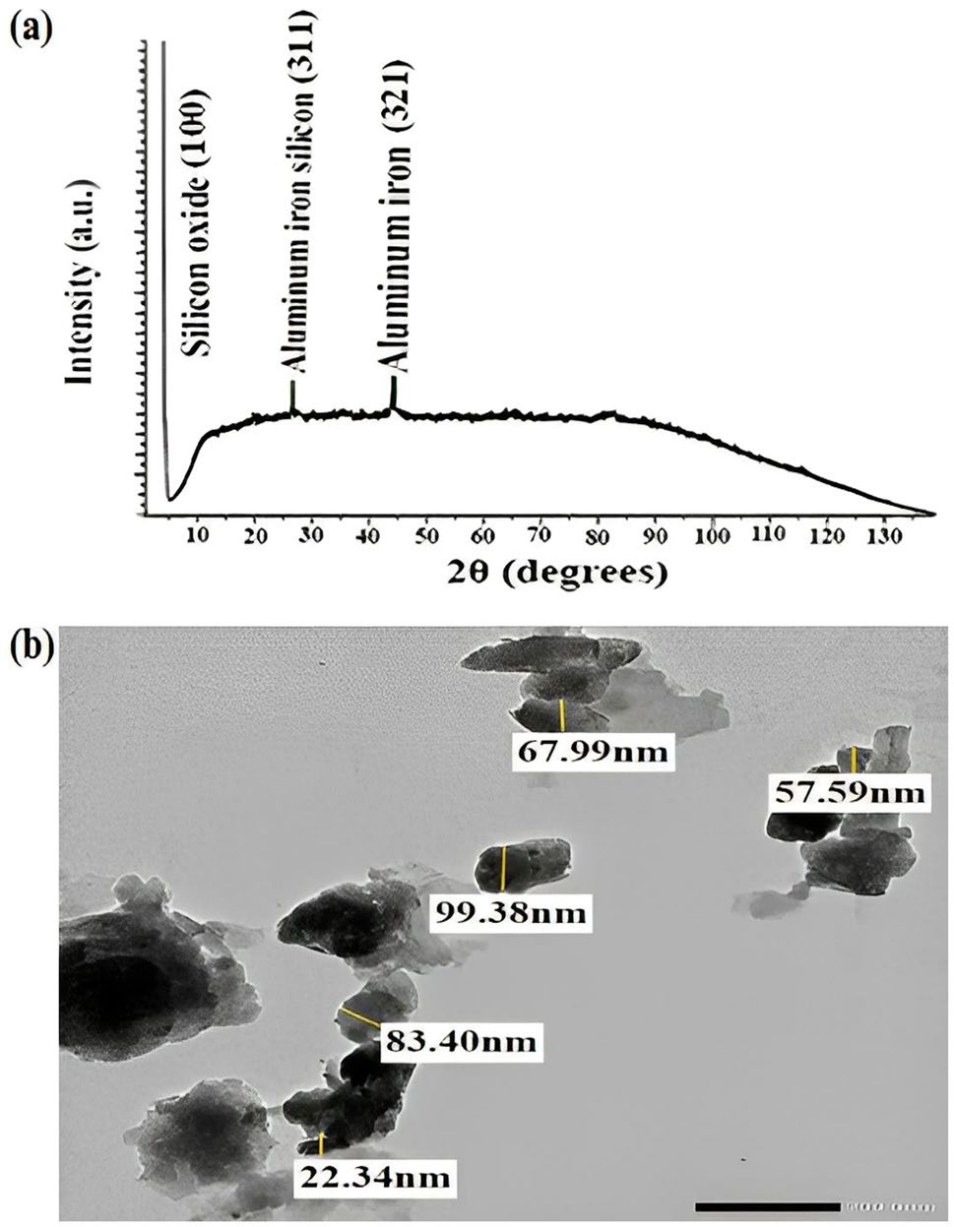
TEM (**a**) and XRD (**b**) analysis of nWTR carrier.

The Diffraction peaks in the XRD pattern for crystal products did not reveal any sharp diffraction characteristic peak over a broad range of d-spacing which evidently demonstrated the poorly ordered particles within the nWTR (Fig. 2a). It is also indicates that, even though the SEM–EDX results highlight the predominance of silicon and aluminum in nWTR, it is likely to be amorphous aluminum and silicon^20,29^. The EDX elemental analysis of nWTR before P, K and Mg sorption shows high percentages of silicon (41.59%) as well as oxygen (22.51%) and moderate fraction of Al (17.99%).

The amorphous nature and abundance of iron and aluminum in nWTRs could have strong impact on P and K adsorption by nWTR. Amorphous Fe and A1 oxides greatly influence the physical and chemical properties of the soil. Ion adsorption, especially, phosphorus (P), ionic charge, swelling and aggregate formation are significantly modified by the presence of amorphous Fe and Al oxides in soil^19,20,30^.

The features and the size of the WTR nanoparticles (nWTR) were explored using TEM analysis. The TEM image of nWTR (Fig. 2b) shows the agglomerated state of nWTR and ascertains nanoscale sizes of nWTR particles (22.34–99.38 nm). The specific surface area and total pore volume of bulk WTR are 53.1 m^2^g^−1^ and 0.020 cm^3^ g^−1^ respectively while nWTR has a specific surface area of 129 m^2^ g^−1^ and a total pore volume of 0.051 cm^3^ g^−1^. The nWTR sample is characterized by approximately 2–3 times larger surface area and total pore volume than bulk WTR samples which demonstrates the high reactivity of nWTR and makes it potential candidate for nutrients adsorption. Similar results were reported by Elkhatib et al.^20^ and Pająk^31^.

#### SEM and EDX analysis

**Figure 3:**
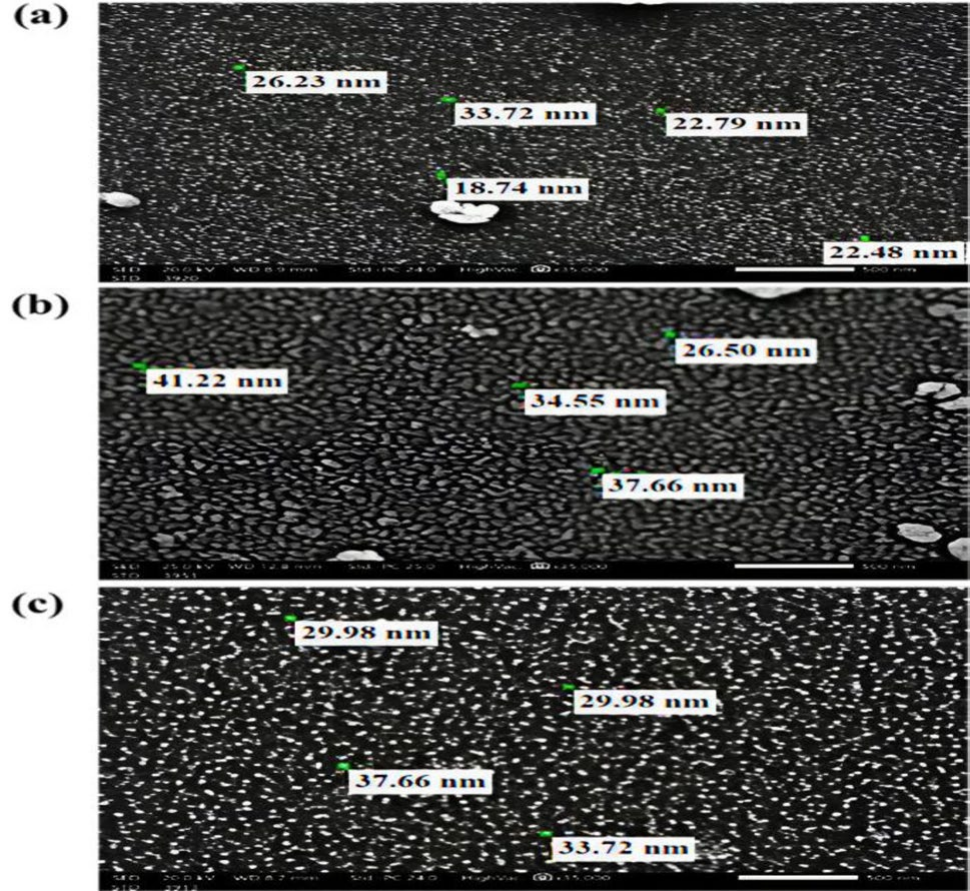
SEM analysis for the synthesized nanoparticles (**A**) nWTR, (**B**) nWTF1 and (**C**) nWTF2.

The SEM and EDX were performed to explore surface characteristics, particles arrangement and element compositions of nWTR before and after loading with two different rates of K, P and Mg nutrients. The SEM image of nWTR before loading with nutrients shows irregular structure with various geometries and sizes in the nanoscale range of 18.74–33.72 nm (Fig. 3a). The SEM images of nWTF1 and nWTF 2 after loading P, K and Mg are presented in Fig. 3b,c respectively. The morphology of nWTF1 and nWTF2 nanoparticles remarkably changed for each nano-fertilizer studied which could be a result of the K, P and Mg adsorption processes^32,33^. The SEM images show clearly coating film of P, K and Mg on the nWTF1 and nWTF2 nanoparticles surface. Meanwhile, the SEM images verified TEM results and affirmed that the nWTF1 and nWTF2 nanoparticles produced are in the nanoscale range. In addition, the SEM image of the nMgO (Fig. S1b) shows that its particle sizes range from 18.30 to 20.92 nm.

The EDX elemental analysis of nWTR before P, K and Mg sorption shows high percentages of silicon (41.59%) as well as oxygen (22.51%) and moderate fraction of Al (17.99%) and comparably low percentages of Ca (10.14%), C (2.64%), Mg (1.89%) and P (1.02%) (Fig. S2a). After loading nWTR with KH_2_PO_4_ and MgO (nWTR1) shows noticeable increases in potassium (41.49%), phosphorus (8.04%) and magnesium (4.69%) and decreases in Si (15.46%) and Al (6.26%) contents in nWTR1 comparative to nWTR as shown in Fig. S2b. Similar trend is noticed with nWTR2 nanostructured fertilizer (Fig. S2c) due to the impregnation of K, P and Mg nutrients in to nWTR structure. The remarkable changes observed in the SEM and EDX analysis indicate the successful loading and adsorption of P–K–Mg nutrients on the nano-carrier (nWTR).

The zeta potential is important measurable indicator of nanoparticles stability and the degree of repulsion between the charged particles in the dispersion. The zeta potential analysis of nWTR is presented in (Fig. S1a).The nWTR demonstrated negative zeta potential value of − 21.3 mV at pH 6.5 which indicates high negative surface charges and electrostatic stability^34^.

#### Fourier transmission infrared (FTIR) measurements

**Figure 4:**
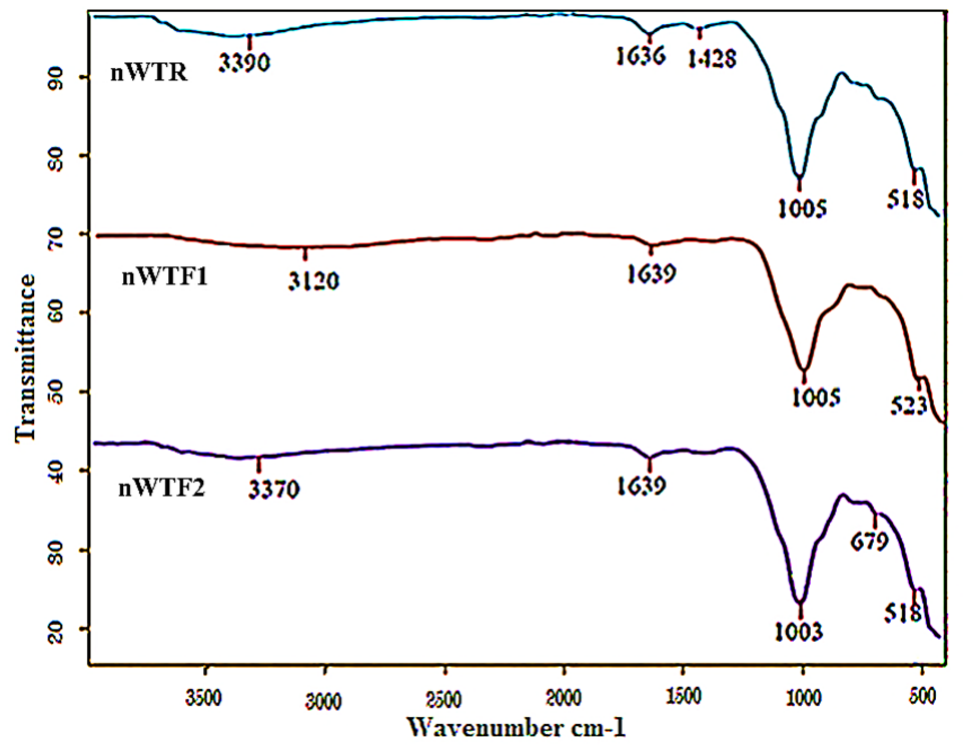
FTIR spectra of nWTR, nWTF1 and nWTF2.

The FTIR spectrum of nWTR presented in Fig. 4 showed characteristic bands at 3390, 1636 and 128 cm^−1^ corresponding to –OH stretching, HO–H bending vibrations and C=C vibrations respectively, with weaker bonds usually vibrate slower than stronger bonds^35^. The observed band at 1005 cm^−1^ is assigned to silicate ions**,** whereas the band that appears at 518 cm^−1^ is associated with Al–O bending vibration^30^. After loading of P, K and Mg onto nWTR, the FTIR spectra of nWTF1 and nWTF2 (Fig. 4) showed bands shift suggesting the interaction of KH_2_PO_4_–MgO conventional fertilizers and nWTRs. The FTIR bands of nWTF1 and nWTF2 spectra (3390, 1636, and 518 cm^−1^) shifted to diverse wave numbers (3120, 1639 and 523 cm^−1^) and (3370, 1639, 1003 and 679 cm^−1^) respectively. In general, higher wave number shifts in FTTR bands are marked by strengthen of the chemical bonds and vice versa. The observed changes in the location and strength of bands at wave numbers range 3390–518 cm^−1^ in nWTF1 and nWTF2 spectra can be attributed to the interaction of KH_2_PO_4_ and MgO with the active functional groups on nWTRs surfaces (i.e.–OH, C–O, Al–O and H bonds)^16,36,37^.

#### X-ray photoelectron spectroscopy (XPS) analysis

**Figure 5:**
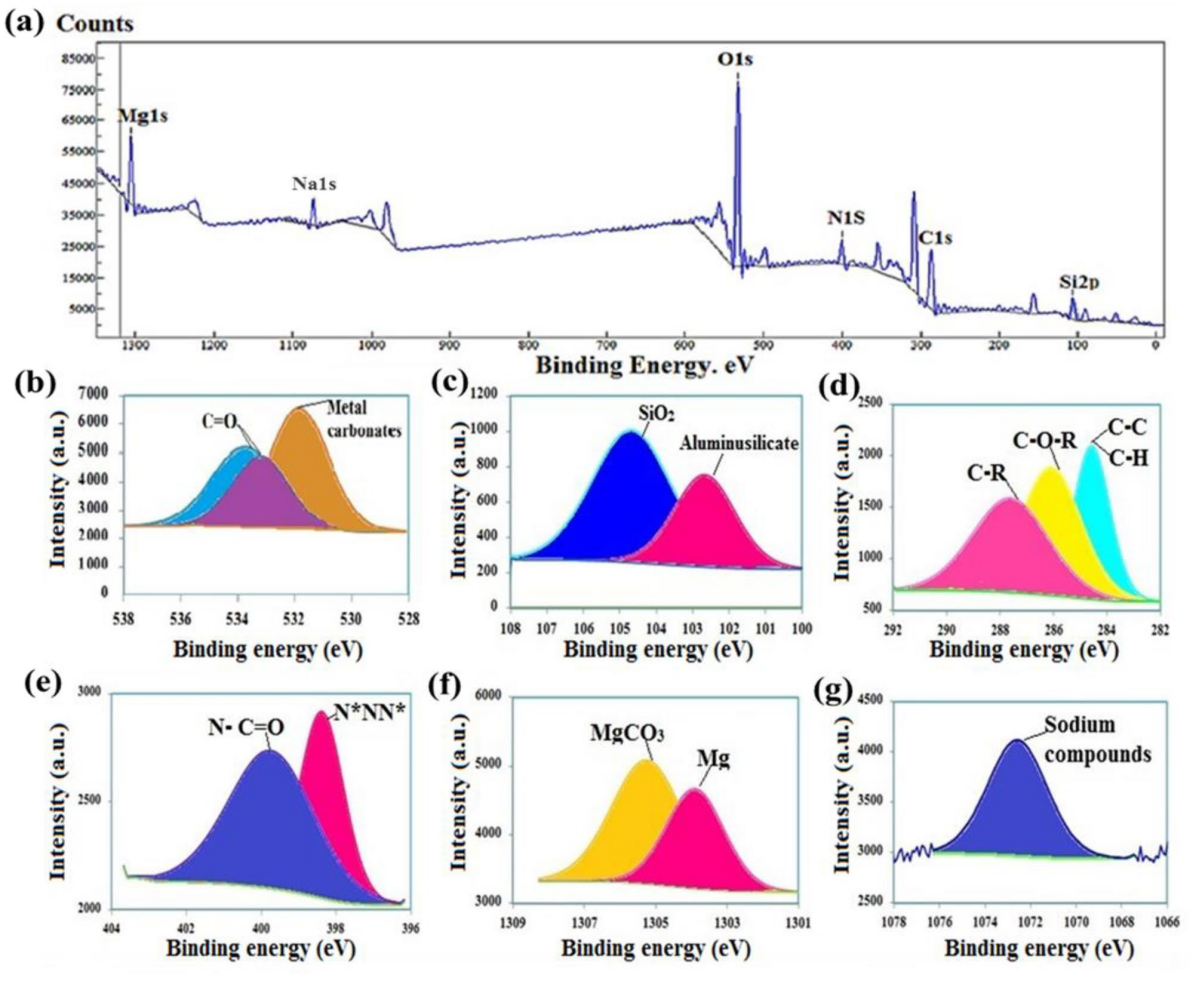
XPS analysis of nWTRs. The XPS scanning spectra of nWTR (**a**) shows six major peaks, XPS high resolution scan of O1s (**b**), Si2p (**c**), C1s (**d**), N1s (**e**), Mg1s (**f**) and Na1s (**g**) region of nWTR.

The chemical states and elemental composition of nWTRs surfaces were investigated using XPS analysis (Fig. 5a). The nWTRs full spectrum showed the existence of Mg, Na, O, N, Cl, and Si. The high resolution of O1s spectra (Fig. 5b) exhibited three peaks at binding energies of 31.84 eV and (533.17, 533.73 eV) assigned to metal carbonates and Organic C=O groups, respectively^38^. The three peaks originated from Si2p at 102.67 and 104.68 eV were indicative of aluminosilicate and silica SiO_2_ groups (Fig. 5c).In addition, three peaks were observed in the high resolution C1s spectra (Fig. 5d) at 284.57, 285.72 and 287 eV due to C–C & C–H, C–O–R and C–R groups respectively ^39,40^. The displayed two peaks of N1s spectra at 398.38 and 399.8 eV (Fig. 5e) were attributed to azide (N*NN*)^40^**,** and N-C=O separately^39^.Furthermore, the tow peaks of Mg1s spectra at 303.9 and 1305.25 eV (Fig. 5f) were assigned to Mg metal and MgCO_3_ respectively^41^, whereas the peak of Na1s spectra at 1072.57 eV (Fig. 5g) was attributed to sodium compounds^40^. As expected, the Si2p, O1s and C1s spectra of nWTR (Fig. 5b–d) confirm the presence of oxygen (O 1s), silicon (Si2p,), and carbon (C 1s) elements which in agreement with the FTIR, XRD and EDS analysis. The integral areas of peaks were normalized and the percentages of bonding groups are shown in Table S3.

#### Possible mechanisms for P–K–Mg adsorption by nWTR carrier

**Figure 6:**
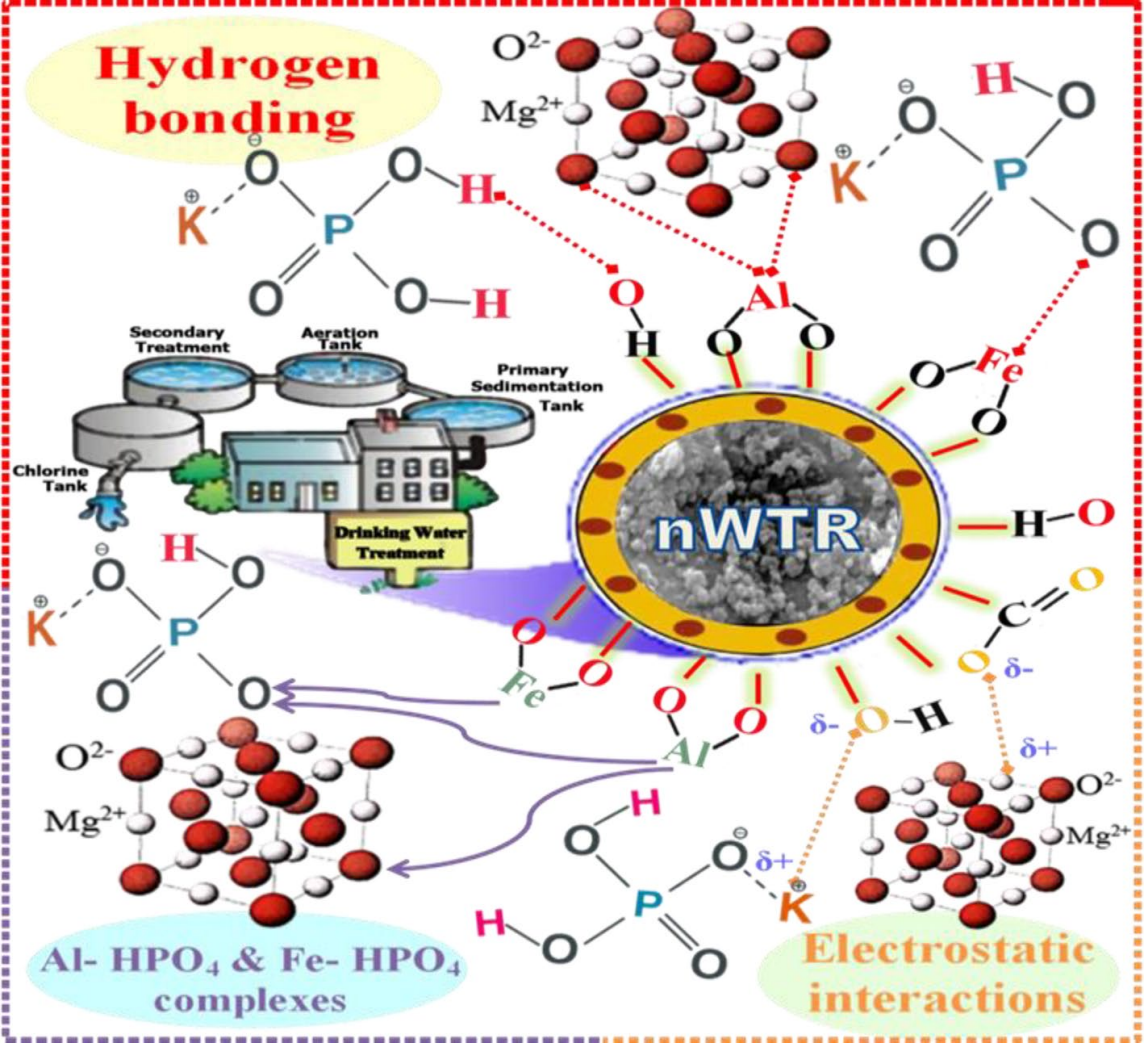
Plausible mechanisms of P–K–Mg adsorption on to nWTR carrier.

The FTIR spectroscopy technique was used to study the interaction between the adsorbates and functional groups on the adsorbent surface. The interpretation of the FTIR is based on the chemical structure of the nWTR before and after loading with K–P–Mg nutrients. Vanishes or shifts of FTIR peaks demonstrate interactions of the adsorbates with functional groups on adsorbents surface. Comparison of nWTRs FTIR spectrum with that of nWTRs loaded with KH_2_PO_4_ + MgO has established the involvement of the functional groups on nWTRs surface (OH, O–Al–O, and Fe hydroxide groups) in the interactivity of nWTRs with the K–P–Mg nutrients. FTIR spectra of nWTR and nWTR-K–P–Mg loaded are shown in Fig. 4. In accordance with XRD, FTIR, and XPS results, the suggested adsorption mechanisms of PO_4_–K–Mg onto nWTR carrier are presented in Fig. 6.The proposed scheme (Fig. 6) shows that the anticipated adsorption mechanisms of PO_4_–K–Mg nutrients by nWTR are: (1) hydrogen bonding (2) electrostatic interaction (3) and Al–HPO_4_ and Fe–HPO_4_ complexes formation**:**

1. *Hydrogen bonding* The produced nWTRs contain hydroxyl group (H-acceptor) whereas the loaded conventional fertilizers (KH_2_PO_4_ + MgO) contain a strongly electronegative hydrogen donor (O). Therefore, hydrogen bonding occurs between H-donor (O) of the fertilizers (KH_2_PO_4_ + MgO) and H-acceptor (OH) of nWTRs. The shift of the absorption peaks at 3390 and 1636 cm^−1^ corresponding to –OH stretching and HO–H bending vibrations might support H-bonding formation during the KH_2_PO_4_ + MgO loading process (Fig. 6).
2. *Electrostatic interactions* The strong affinity of the function groups of nWTR (–OH, C–O and C=O) for cationic nutrients species of nWTR1 & nWTR2 (K, Mg) greatly indicates electrostatic interactions participation in K and Mg adsorption process (Fig. 6).
3. The Al–HPO_4_ and Fe–HPO_4_ complexes formation is quite possible through binding Al and/or Fe of nWTRs to O atom of KH_2_PO_4_ + MgO (3) (Fig. 6).

### Nano-fertilizer parameters and release studies

#### Water-retention (WR) behavior of the nano-enabled fertilizer (NEF) in soil

**Figure 7:**
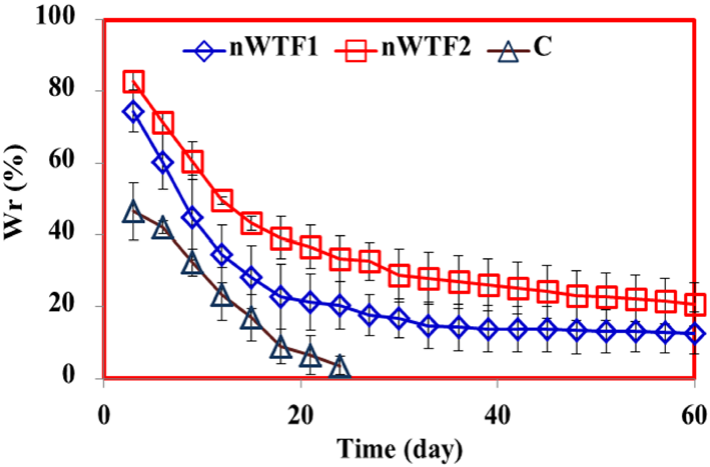
Water retention behavior of soil without and with nano-enabled fertilizers (nWTF1 and nWTF2). Error bars correspond to mean ± standard error of the mean.

The nano enabled fertilizer (NEF) is capable of conserving soil moisture when the material of the nano-carrier is good water absorbent. The water preserved by the carrier during irrigation can be progressively released to the soil to dissolve the mineral nutrients in the nano-carrier for effective release to the plants via diffusion and to fulfill the plant water demands in drought-prone areas^42^. The WR behavior of soil with and without nano-enabled fertilizers (NEF) was evaluated against time. Figure 7 shows that addition of NEF (nWTF1 and nWTF2) to the soil increased its water retention performance. The WR in the control soil was about 47% on the 5th day, reached 3.6% on the 24th day, whereas, the WR of the soil mixed with nWTF1 and nWTF2 was 74.40% and 83.01% on the 5th day and reached 20.41% and 33.38% on the 24th day respectively. After 26 days, the water content of the control soil was almost evaporated whereas nWTF1 and nWTF2 fertilizer treatments showed WR values of 12.70% and 20.69% respectively after 60 days. It is quite clear that the nano-enabled fertilizers used effectively improved water retention capacity of the soil and slowed down water evaporation with nWTF2 being the most efficient due its highest content of nWTR^43^. Therefore, nWTF2 can extend irrigation cycles, and enhance drought tolerance of plants. Similar results have been reported by using slow-release urea fertilizer based starch and hydrogel^44^.

#### Release behavior of nano-enabled fertilizer (NEF) in water

**Figure 8:**
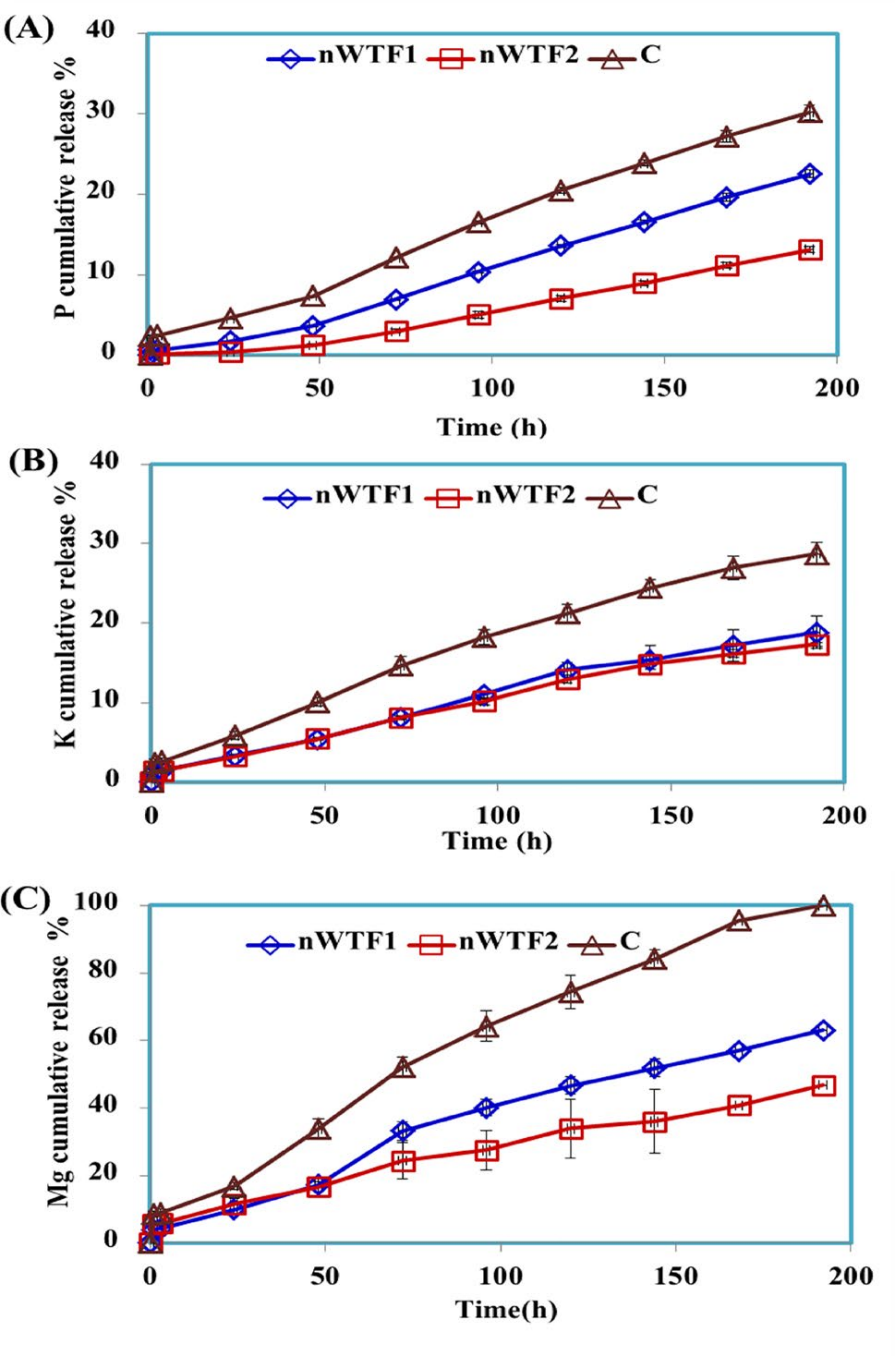
Cumulative release of P (**A**), K (**B**) and Mg (**C**) from control and NEF in water.

Nutrient release curve is a key feature characterizing the nutrients efficient release performance of NEF. The release behaviors of P, K and Mg from the prepared NEF were examined in water. Figure 8a–c shows that the release rates of P, K and Mg from control (standard sources, KH_2_PO_4_ _**+**_ MgO_***NP***_) were much faster than the released rates of P, K and Mg from the NEF. The released rates of P from control, nWTF1 and nWTF2 in water reached 30.24%, 22.56% and 13.14% respectively within 192 h (Fig. 8a). For K release, the cumulative rate of K released from C was about 28.74% within 192 h in distilled water (Fig. 8b) as compared to 18.82% and 17.79% of K released from nWTF1 and nWTF2 respectively within the same time frame. For Mg release, the Mg cumulative percentage released from control was about 100% within 192 h in distilled water whereas, the cumulative release percentages from nWTF1 and nWTF2 at the same time interval were 63.11% and 46.79% respectively (Fig. 8C). The long duration of P–K–Mg released from tested fertilizers increased with increasing nWTR content. These results demonstrate the potential use of nWTF1 and nWTF2 as promising sustained release fertilizers with nWTF2 being the most efficient.

#### Nutrients release behavior of nano-enabled fertilizer (NEF) in soil

**Figure 9:**
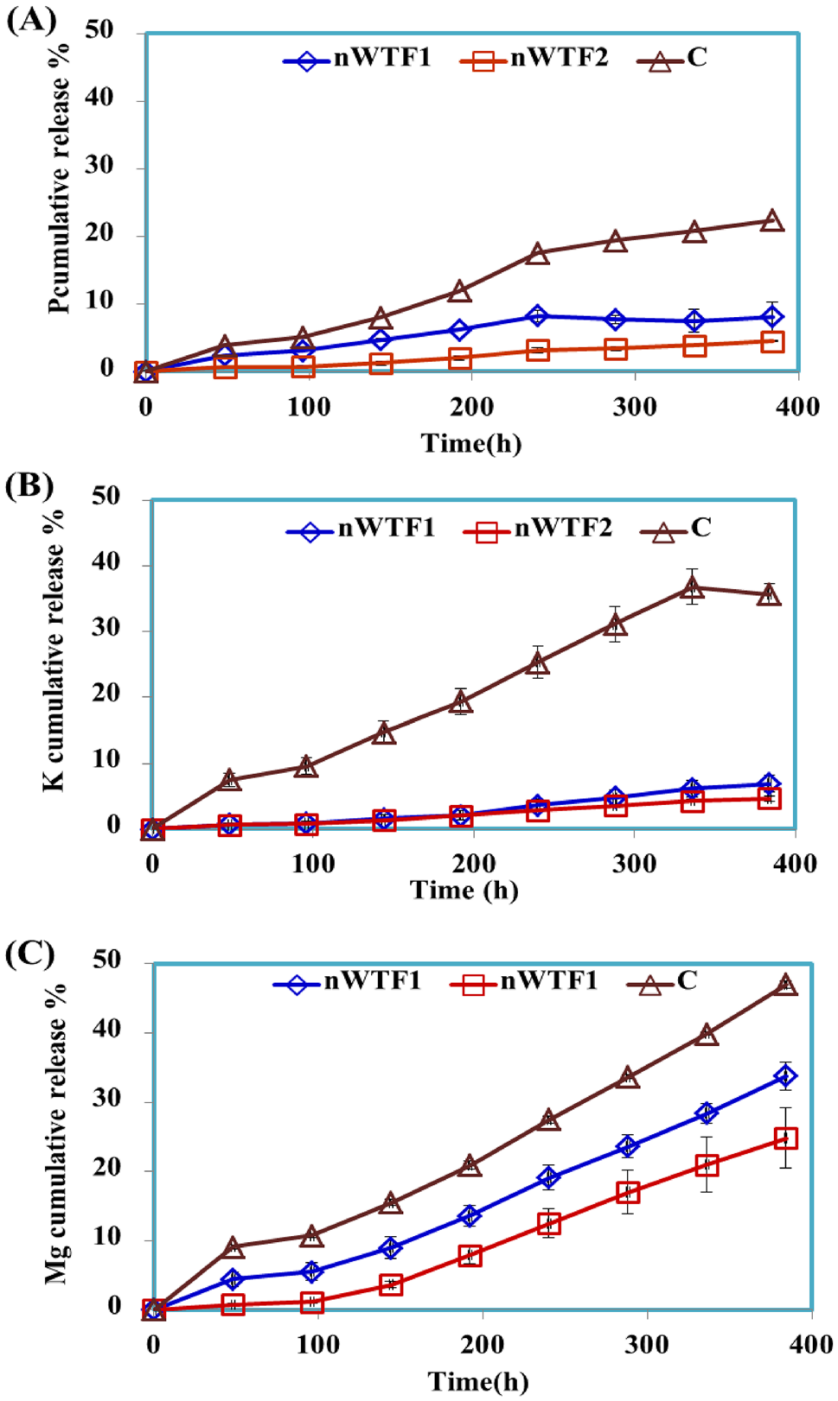
Cumulative release of P (**A**), K (**B**) and Mg (**C**) from control and NEF in soil.

The release behaviors of P–K–Mg from control and prepared NEF were examined in soil column experiment and the results are presented in Fig. 9. The nutrients studied (P–K–Mg) were added to the sandy soil in the form of NEF (nWTF1& nWTF2) to improve nutrients availability to plants through alleviating nutrients loss by leaching and enhancing soil nutrient retention^45–47^.

The results in Fig. 9 show that the released rates of P, K and Mg from control, nWTF1 and nWTF2 in soil were much lower than those in soil. The phosphorus cumulative release percentages from control, nWTF1 and nWTF2 in soil have reached 22.41%, 8.12 and 4.51% respectively within 384 h (Fig. 9a). Meanwhile, the cumulative ratio of K released from control was about 35.71% within 16 days (384 h.) in soil leachate as shown in Fig. 9b which is almost 5 and 7.7 times higher than that of nWTF1 (6.90%) and nWTF2 (4.65%) respectively. Moreover, The Mg cumulative percentage released from control in soil leachate was about 47.01% within 384 h, whereas the cumulative release percentages of Mg from nWTF1 and nWTF2 at the same time interval were 33.78% and 24.82% respectively, shown in Fig. 9c. Remarkably, the cumulative release percentages of P, K and Mg in soil leachate were in the following order: Classical fertilizers >> nWTF1 > nWTF2. It is therefore concluded that nWTF2 exhibits a preferable sustained-release property and is considered a promising efficient and sustainable alternative to substitute classical P, K and Mg fertilizers. In addition to the encouraging features of using nWTF2 rather than classical fertilizers to deliver nutrients, the nWTR carrier used is safe, eco-friendly, and adaptable to soil, plants, and other organisms. The excellent P–K–Mg efficient-release achievement is accredited to the characteristics of the nWTR carrier, including high water retention and electrostatic attraction for K and Mg, the interaction between potassium hydrogen phosphates and Al & Fe oxyhydroxides groups on the surface of nWTR and formation of (Al- and Fe) complexes as well as H-bonds formation during the KH_2_PO_4_ and MgO loading process. Elkhatib et al.^16^ reported that very small percentage (< 3.5%) of P was released from P-saturated nWTRs demonstrating P stability in P-nWTR system. The excellent adsorption capability of nWTR for inorganic pollutants indicates its potential applicability as a carrier for plant nutrients.

#### Kinetics and modeling of P, K, and Mg release from NEF in water and soil

In the present study, the released data obtained from leaching experiments in water and soil were used to assess the suitability of various kinetic models (first order, Elovich, parabolic diffusion and power function) to describe the kinetics of P, K and Mg release from water and soil amended with NEF. To obtain proper information from kinetic models and their parameters, the used models must properly fitted to the experimental data. If results are fitted with lower errors, the release process can be better interpreted. Quality fitting can only be achieved in linear and nonlinear models by calculating the standard error of estimates (SE) for the models used. Thus the validation of the used kinetic models was based on determination coefficient (R^2^) and standard error of estimate (SE)^17,27^.

**Figure 10:**
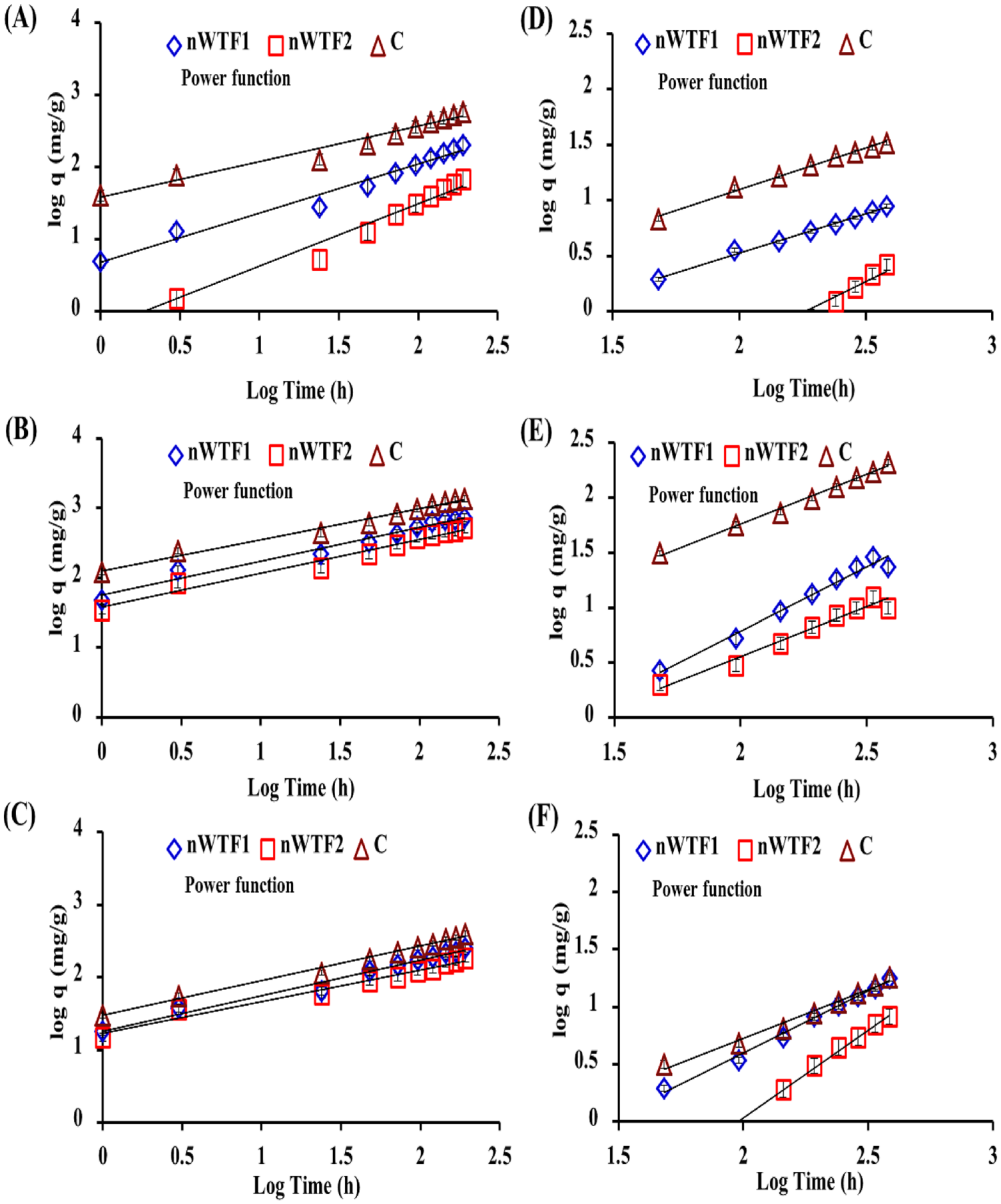
Release kinetics model of P (**A**), K (**B**) and Mg (**C**) in water and P (**D**), K (**E**) and Mg (**F**) in soil.

The experimental release data of P, K, and Mg from NEF in water and soil were modeled using four kinetic models to understand macronutrients release kinetics and to predict the release rate. The four kinetic models tested and its parameters together with R^2^ and SE values are displayed in Tables 1, 2 and 3. The experimental data of P, K, and Mg best fitted the power function model with the determination coefficient (R^2^) between 0.964 and 0.995 and SE between 0.017 and 0.115 for P, R^2^ between 0.967 and 0.993 and SE between 0.017 and 0.115 for K and (R^2^) between 0.938 and 0.992 and SE between 0.028 and 0.066 for Mg. The power function model has shown the lowest SE values which indicates that this model is the most suitable model for describing the P, K and Mg kinetics release data from NEF and conventional fertilizers in water and soil (Tables 1, 2, 3 and Fig. 10). In the power function model the reversibly P, K and Mg adsorbed phases are mainly related to the initial concentration of P, K and Mg and are proportional to the fractional power of time and controlled by the desorption mechanism^48–50^. The values of apparent desorption rate coefficients (K_d_) of power function model for P, K and Mg are presented in Tables 1, 2 and 3. In general, desorption rate coefficient “K_d_” values of conventional fertilizers were the highest in comparison with NEF. For all the studied treatments, P, K and Mg release followed the order: conventional fertilizers > nWTF1 > nWTF2. The difference in the effect of studied treatments on macronutrients release is related to the amount of nWTR content in the prepared NEF. In our previous studies^20^, we have shown the high P adsorption capability of nWTR. Approximately 95% of P was rapidly adsorbed within 100 min of adsorption forming chemically stable phosphate phases (inner-sphere complexes) and facilitating slow P removal from the systems. The low K_d_ values obtained from nWTF2 in the current study suggests its use as a very promising and practical solution to improve fertilizer use efficiency.

#### Comparison of P and K sustained release from nWTR fertilizers and the other fertilizers

The release of P and K from nano enabled fertilizers (nWTF 1 and nWTF2) is compared with different fertilizers reported in literature (Table 4). It can be seen that the nWTF2 outperformed all the listed fertilizers in controlling P and K release from soil and also outperform most of the listed fertilizers in controlling P and K release in water.

**Table 4:** Sustained release of P and K from nWTR fertilizers and the other fertilizers reported in literature.

Fertilizers	Sustained-release performance	References
nWTF1	within 384 h were 8.1% and 6.9% respectively in soil	Present study
nWTF2	P and K release within 384 h were 4.5% and 4.7% respectively in soil	Present study
Mg-enriched biochar	P release within 50 h was less than 20% in soil	^40^
Biochar embedded semi-IPN based SRF	The release ratios of P and K within 30 d were less than 80% in soil	^51^
Bentonite modified biochar P SRF	P release within 15 d was 72.6% in soil	^52^
nWTF1	P and K release within 192 h were 30.24% and 18.82% in water	Present study
nWTF2	P and K release within 192 h were 22.56% and 17.79% in water	Present study
Biochar/struvite composites as N and P fertilizer	The cumulative release of P after 84 d was 6.84% in distilled water, and 59.12% in citric acid solution	^53^
P-enriched biochar fertilizer	The extractable P in water and citric acid reached 52% and 61% after 5 d	^54^
Blended biochar	Total water-soluble accumulative P was 44.6 mg g^−1^ after 84 d	^55^
Biochar-based P fertilizers	Total P release 6.47% ((coffee husk)) and 8.99% (poultry litter) within 1 h in water	^56^
Biochar-based P SRF	Bioavailable P release was 40% after 5 d in water	^57^

#### Plant growth parameters

**Figure 11:**
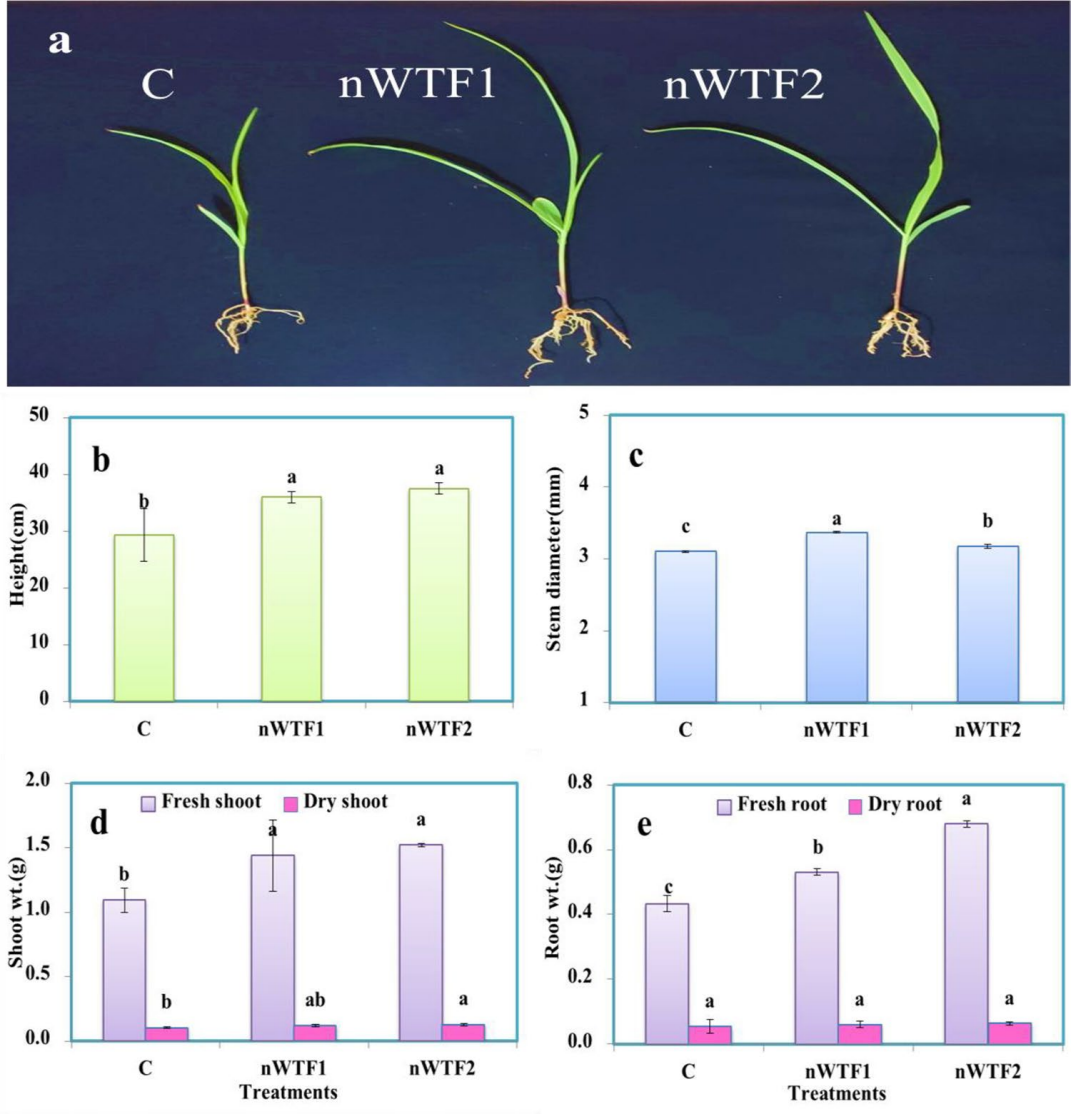
(**a**) Picture of *maize* (corn) plants at 25 days (**b**) plant height (**c**) stem diameter (**d**) shoot fresh and dry weights (**e**) root fresh and dry weights.

**Figure 12:**
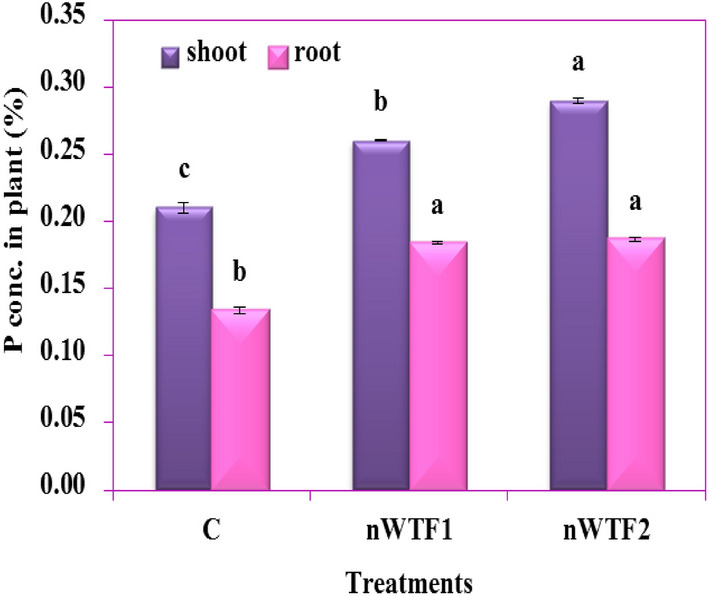
Phosphorus concentration in plant shoot and root tissues.

Data presented in Fig. 11 displayed that application of nano-enabled fertilizers (nWTF1and nWTF2) can have a significant impact on growth parameters of maize plants as compared to the commercial fertilizers (control). Both nWTF1and nWTF2 treatments gave the highest values of plant height (36 cm and 37.5 cm) respectively, whereas the lowest value (29.27 cm) was recorded with control (Fig. 11b). Additionally, nWTF1and nWTF2 treatments exhibited the highest values of shoots fresh, shoots dry and roots fresh weights (1.44 g and 1.52 g), (0.12 g and 0.13 g) and (0.53 g and 0.68 g) respectively as compared to control (1.10 g, 0.11 g and 0.43 g) respectively (Fig. 11d,e). In contrast, the highest value of stem diameter was recorded with nWTF1 treatment (3.37 mm) followed by nWTF2 treatment (3.17 mm) (Fig. 11c). The phosphorus content in maize shoot and root tissues under nWTF2 treatment achieved the highest P concentrations in shoots (0.29%) and in roots (0.187%), followed by nWTF1 treatment (0.26% in shoots and 0.184% in roots). The lowest P content in shoots (0.21%) and in roots (0.13%) was recorded in control treatment plants (Fig. 12a). The use of nWTF1and nWTF2 proved to be effective in improving plant growth and significantly (*p* < 0.05) increased phosphorus content in maize plants (Figs. 11 and 12).

Our results are consistent with Hassani et al.^58^ who reported that application of nano-fertilizer to peppermint (*Mentha piperta* L.) resulted in the greatest number of branches and leaves, highest wet and dry weight of leaves, wet and dry weight of stems and wet and dry weight of plant. As described by Usman et al.^59^**,** the use of nano fertilizers lowers eutrophication, allows fertilizers to reach their intended location more efficiently, accelerates seed germination which resulting in a high yield in a short period of time. Thus nano-fertilizers are considered as smart delivery systems with distinct role in crop production.

In summary*,* this study demonstrates the potential use of the nano-enabled fertilizer (nWTF1and nWTF2) for enhanced plant growth and sustainable controlled release of nutrients. The use of nWTR as a carrier in the produced NEF not only reduces waste but also provides an environmentally friendly alternative to traditional chemical fertilizers and can additionally be utilized to manufacture value-added products as climate-smart environmental solution. Further studies are needed to investigate its long-term effect on crop yield and to optimize the preparation of nano-enabled fertilizers accordingly.

## Conclusion

Promising nano-enabled fertilizers (nWTF1 and nWTF2) were produced by impregnation of nanostructured water treatment residual (nWTR) with (KH_2_PO_4_ + MgO) at 1:1 and 3:1 (w/w) ratios respectively using a planetary ball mill. Application of the produced nano-fertilizers to the sandy soil effectively improved the capacity of the sandy soil to retain water during the 60 days study with nWTR2 being the most efficient. The water content of the control soil was almost evaporated after 26 days, whereas the soil treated with nWTF2 fertilizer showed water retention value of 20.69% after 60 days. The leaching behavior study demonstrated that nWTF2 significantly controlled P–K–Mg nutrients release due to its lowest leaching losses of P–K–Mg (4.5–4.7–24.8%) respectively in comparison with nWTR1 (8.1–6.9–33.8%) and classical fertilizer (22.4–35.7–47%). These results showed that the nano-enabled fertilizers exhibited a sustained release of nutrients, with a gradual decline in the release rate over time. This is a significant advantage over traditional fertilizers, which release nutrients quickly and can lead to leaching and nutrients losses. The low standard error (SE) values of power function model verified its high potentiality to predict NEF release data and ascertained that the reversibly P, K and Mg adsorbed phases were mainly related to the initial concentration of P, K & Mg and were proportional to the fractional power of time. The obtained results from pilot experiment on zea maize plants revealed that the nano-enabled fertilizers (nWTF1 and nWTF2) significantly promoted growth, and P content compared with the commercial chemical fertilizer treated plants. The pilot experiment was carried out to evaluate the effect of the proposed NEF on growth and nutrient (P) content of maize plants within a short time period of 25 days after fertilizers application. We highly recognize that field trials are necessary to investigate the long-term impacts of the proposed NEF on growth and productivity of corn and other plants. Therefore, long term future studies at pot and field scale should be conducted to assess plant productivity, nutrients availability together with their environmental impacts.

### Supplementary Information

Supplementary Information.

## Data Availability

The datasets used and/or analyzed during the current study can be available from the corresponding author upon reasonable request.
