# Supplementary material for: Novel nano-fertilizers derived from drinking water industry waste for sustained release of macronutrients: performance, kinetics and sorption mechanisms
Source: Sci Rep. 2024 Mar 8;14:5691. doi: 10.1038/s41598-024-56274-0 (PMC10920638; doi:10.1038/s41598-024-56274-0)
Supplement: Supplementary file 1 — Supplementary Information. [file 41598_2024_56274_MOESM1_ESM.docx]

**[Novel nano-fertilizers derived from drinking water industry waste for sustained release of macronutrients: Performance, kinetics and sorption mechanism](https://www.sciencedirect.com/science/article/pii/B9780128213544000054)s**

**Samira S. Elsabagh^1^, Elsayed A.Elkhatib^2^*, Mohamed Rashad^1^**

^1^Arid Lands Cultivation Research Institute, City of Scientific Research and Technological Applications, New Borg El-Arab, 21934, Alexandria, Egypt

^2^Department of Soil and Water Sciences, Faculty of Agriculture (El-Shatby), Alexandria University, Alexandria 21545, Egypt

**Supplemental Materials**

**WTRs characteristics**. The chemical and physical characteristics of the WTRs used to produce NEF are presented in Table S1. High OM, high CEC and high WHC with low EC and low nutrients content were observed in the WTRs.

**Table S1. Physical and chemical characteristics of WTRs.**

| **Characteristics** | **WTRs**^a^ |
| --- | --- |
| pH | 7.4 ± 0.1 |
| EC dS m^-1^ | 1.6 ± 0.03 |
| Real density g cm^-3^ | 1.82±0.71 |
| Bulk density g cm^-3^ | 0.71±0.11 |
| OM g kg^-1^ | 61.0 ± 2.3 |
| CEC Cmol(+‏)kg^-1^ | 341.4 ± 0.4 |
| WHC g kg−1 | 470.0±2.18 |
| Total Elements |  |
| N g kg^-1^ | 3.2 ± 0.1 |
| P g kg^-1^ | 2.0 ± 0.1 |
| K g kg^-1^ | 2.3 ± 0.3 |
| Mg g kg^-1^ | 0.24± 0.03 |
| Al g kg^-1^ | 36.1 ± 2.9 |

EC electrical conductivity, OM organic matter, CEC cation exchange capacity, WHC water holding capacity .

^a^ Means of three samples ±SD**.**

[D. L. Sparks](https://books.google.com.eg/ebooks?output=ws2&as_brr=5&q=inauthor%3A%22D.%20L.%20Sparks%22&hl=en) etal.2001, [Methods of Soil Analysis.](https://books.google.com.eg/books?id=pe3wAAAAMAAJ&source=gbs_similarbooks)  Soil Science Society of America, Madison, Wisconsin, USA .

**Soil Location and Sampling.** Soil samples were collected from the rear of the 0-30 cm from EL- Alamien, Alexandria, Egypt, and transported into the laboratory.

**Soil characteristics**. The chemical and physical characteristics of the soil used in controlled release studies are presented in Table S2. The soil was sandy, moderately alkaline (pH 8.2) with low OC, EC and low nutrients content.

**Table** S2**.** The physical and chemical characterization of the tested soil

| **Soil parameter** | **Sandy soil** |
| --- | --- |
| pH | 8.20 |
| EC (dS/m) | 0.61 |
| Total N (%) | 0.003 |
| Available P (mg/kg) | 5.34 |
| Organic C (%) | 0.044 |
| Sand (%) | 92.96 |
| Silt (%) | 0.21 |
| Clay (%) | 6.83 |
| Texture | Sandy |
| **Soluble ions (mg kg^-1^)** |  |
| Ca^2+^ | 51.60 |
| Mg^2+^ | 11.76 |
| Na^+^ | 55.20 |
| K^+^ | 3.90 |
| CO_3_^2-^ | 0.00 |
| HCO_3_^-^ | 109.80 |
| SO_4_^-^  Cl^-^ | 12.48  142.00 |

[D. L. Sparks](https://books.google.com.eg/ebooks?output=ws2&as_brr=5&q=inauthor%3A%22D.%20L.%20Sparks%22&hl=en) etal.2001, [Methods of Soil Analysis .](https://books.google.com.eg/books?id=pe3wAAAAMAAJ&source=gbs_similarbooks)  Soil Science Society of America, Inc.Madison, Wisconsin, USA.

**Table** S3**.** XPS analysis of nWTR.

| **Name** | **Peak BE** | **FWHM eV** | **Area (P) CPS.eV** | **Atomic %** | **Q** |
| --- | --- | --- | --- | --- | --- |
| **O1s** | 528.53 | 4.41 | 238576 | 34.57 | 1 |
| **Mg1s** | 1292.11 | 3.65 | 76714.3 | 7.88 | 1 |
| **C1s** | 284.24 | 5.05 | 85451.1 | 31.59 | 1 |
| **N1s** | 396.07 | 4.04 | 35942.6 | 7.83 | 1 |
| **Si2p** | 103.97 | 4.71 | 26814.4 | 11.15 | 1 |
| **Na1s** | 1062.51 | 3.84 | 36334.3 | 2.99 | 1 |

**Table** S4**.** The P, K and Mg contents of nWTR (nano-carrier ) and sustained release fertilizers ( nWTF1 and nWTF2**).**

|  | P (mg/g) | K(mg/g) | Mg(mg/g) |
| --- | --- | --- | --- |
| nWTR | 6.07± 0.014 | 7.94± 0.03 | 2.4± 0.17 |
| nWTF1 | 113.9 ± 0.272 | 143.40 ± 1.08 | 60.0± 2.60 |
| nWTF2 | 57.00 ± 0.114 | 71.70 ± 0.07 | 31.0± 0.01 |

Means of three samples ±SD

**Water-retention behavior of nWTF1 and nWTF2 in soil.**  A sandy soil was selected for the water retention test because the productivity of sandy soils is limited by their low water holding capacity, high water percolation losses and low fertility status,. Thus enhanced fertilizers and water use efficiency of sandy soils are of paramount importance in sustained agricultural development (Huang and Hartemink, 2020; Yost and Hartemink, 2019; Huang et.al.2019). About 1 g of the dried (NF) samples was added into 100 g of dried sandy soil in a 250 mL of beaker . At the same time, 100 g of dried sandy soil without (NF) was placed in another beaker as a control. Then each beaker was added 50 mL distilled water and weighed. The beakers were covered by aluminum sheet at experiment time, all beakers weighed once every three days at room temperature until they got to constant mass. The water-retention behavior WR (%) of the soil was calculated by Eq. (1):

w_r_= w_t_−w/w_0_−w X100% (1)

Where w_r_ is water-retention rate and W is the total mass of the sandy soil and beaker.w_0_ is the total mass of sand, beaker, and sample after adding distilled water. w_t_ is the total mass of sand, beakers and samples at regular intervals (Wei et al.2019).

**Controlled release behavior of NEF.** The release pattern of nWTF1 and nWTF2 NF were first studied in distilled water. Briefly, 0.1 g of dried sample and 10 mL of distilled water was added into a dialysis bag, which then was transferred to an Erlenmeyer flask containing 200 mL of distilled water. 10 mL of the solution has been withdrawn from the flask at preset time intervals and then 10 mL of fresh distilled water was added to maintain the volume of the media constant. The P, K and Mg content in the solution was determined by ultraviolet spectrophotometry/flame photometry accord to the standard curve made in advance. Herein, the slow release behavior of nWTF1 and nWTF2 NF were also studied in soil. Briefly, PVC pipes with a diameter of about 8 cm, and a height of about 30 cm, and filter paper with 200-mesh-nylon-cloth–sealed bottom were used to contain the mixture of 1 g dried NF and 300 g of dried sandy soil. After-wards, 70 mL of distilled water was added to each soil column every 48 hrs. At preset intervals, 10 mL of soil leaching solution was collected and the P, K and Mg content in the aqueous solution was measured by ultraviolet spectrophotometry. The measured values in soil and water were compared with nutrient release patterns of the soil treated with P, K and Mg conventional fertilizers as a control(Wei et al.2019)^22^**.** The release values in soil and water were obtained under the same experimental conditions.

The amount of released P, K and Mg per g of fertilizers [q (mg/g)] in water and soil were calculated using the following Eq. (2) reported by Qian et al.2013) ^23^:

q =Ci*V/mb (2)

Where Ci is the P, K, Mg concentration (mgl^-1^), V is the volume of water (L), and mb (g) is the mass of the nWTR1 and nWTR2 and C added in to water.

The cumulative release Cr(%) will be calculated by Eq. (3) reported by Wei et al. ^22^.

C_r_ =V_E_∑^n−1^C_i_ +V_0_Cn/M_0_ X 100% (3)

Where C_r_ is the cumulative release rate, V_E_ and V_0_ are the initial volume of the sample volume and release medium, respectively. C_i_ and C_n_ are the fertilizer concentrations, i and n are the sampling times, M_0_ is the total mass of the fertilizer loaded in the sample.

**
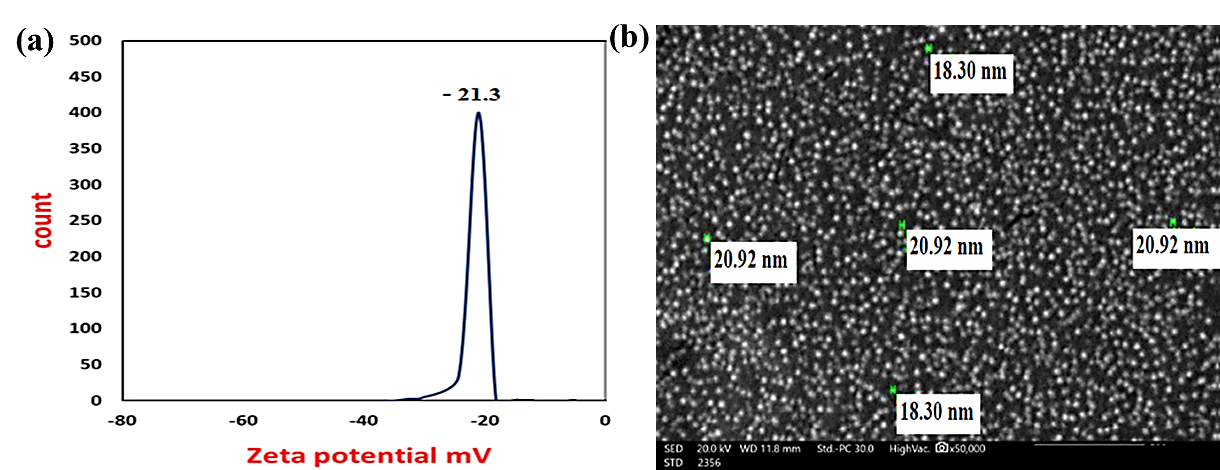
**

**Figure S1.** Zeta potential of nWTR at pH 6.**5** (a) and SEM image of MgO NPs (b)


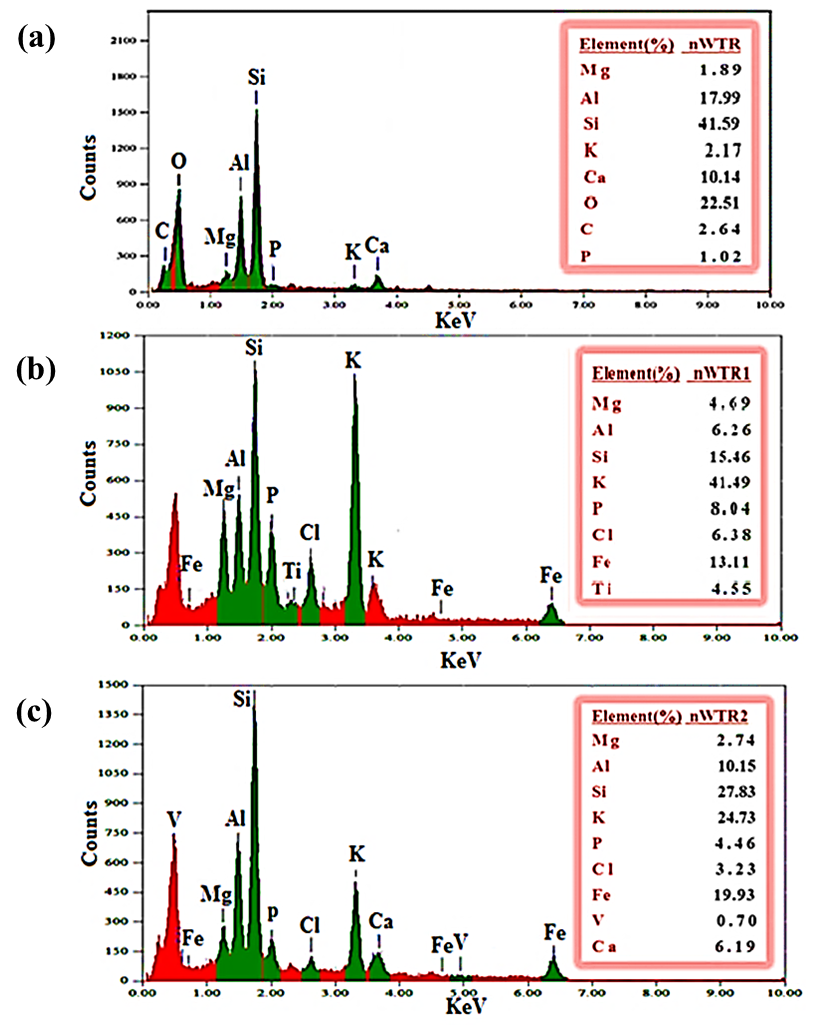


**Figure S2.** EDX analysis for the synthesized nanoparticles (a) nWTR, (b) nWTF1 and (c) nWTF2

**REFERENCES**

Huang, J., Hartemink, A. E.. Soil and environmental issues in sandy soils. Earth-Science Reviews 208 , 103295(2020)

Huang, J., Hartemink, A.E., Arriaga, F., Chaney, N.W.,. Unraveling location-specific and time-dependent interactions between soil water content and environmental factors in cropped sandy soils using Sentinel-1 and moisture probes. J. Hydrol. 575,780–793 (2019).

Qian, T., Zhang, X., Hu, J. & Jiang, H. Effects of environmental conditions on the release of phosphorus from biochar. Chemosphere. **93,** 2069-2075 (2013). http://dx.doi.org/[10.1016/j.chemosphere.013.07.041](http://dx.doi.org/%2010.1016/j.chemosphere.013.07.041).

Wei, H., Wang, H., Chu, H. & Li J. Preparation and characterization of slow-release and water-retention fertilizer based on starch and halloysite. Int. J. Biol. Macromol. **133**:1210-1218(2019)**.** <https://doi.org/10.1016/j.ijbiomac.2019.04.183>.

Yost, J.L., Hartemink, A.E. Soil organic carbon in sandy soils: a review. Adv. Agron. 158, 217–310 (2019).
